# Identification and Quantitation of Ursolic and Oleanolic Acids in *Ilex aquifolium* L. Leaf Extracts Using ^13^C and ^1^H-NMR Spectroscopy

**DOI:** 10.3390/molecules24234413

**Published:** 2019-12-03

**Authors:** Doreen Palu, Ange Bighelli, Joseph Casanova, Mathieu Paoli

**Affiliations:** Université de Corse-CNRS, UMR 6134 SPE, Equipe Chimie et Biomasse, Route des Sanguinaires, F- 20000 Ajaccio, France; palu_d@univ-corse.fr (D.P.); bighelli_a@univ-corse.fr (A.B.); joseph.casanova@wanadoo.fr (J.C.)

**Keywords:** *Ilex aquifolium*, solvent extracts, component identification, quantitative ^1^H-NMR, ursolic acid, oleanolic acid

## Abstract

Leaves of *Ilex aquifolium* L. have been used for their therapeutic properties. In previous studies, components contained in the leaves were first isolated by various chromatographic techniques. Then, quantitation of oleanolic and ursolic acids, which are responsible for the biological and therapeutic activities of the plant, was performed by HPLC, HPTLC, and somewhat by GC-MS. Our objective was to develop a simple method that allows the identification of compounds contained in the leaves of Corsican *I. aquifolium* and to quantify ursolic and oleanolic acids. Leaves were successively extracted with hexane and dichloromethane. The extracts were chromatographed on silica gel and the fractions of column chromatography submitted to ^13^C-NMR analysis, following a computerized method developed in the laboratory. ^13^C-NMR allowed the identification of various triterpenes including ursolic acid and oleanolic acid. Quantitation of both acids was achieved, for the first time, by ^1^H-NMR after validation of the method (accuracy, precision, linearity, limit of detection and limit of quantitation). Ursolic and oleanolic acids accounted for 55.3% and 20.8% of the dichloromethane extract, respectively. This represents 1.3% and 0.5% of the mass of dried leaves. ^1^H-NMR spectroscopy appeared as a powerful tool for a rapid quantitation of biologically active compounds from *I. aquifolium*.

## 1. Introduction

Recognizing known substances by analytical methods is widespread in the flavor and fragrance industry as well as in the pharmaceutical industry. For instance, identification of volatile components contained in essential oils is routinely achieved by gas chromatography-mass spectrometry (GC-MS) in combination with retention indices (RIs) on two chromatographic columns of different polarity. Identification of heavy compounds in solvent extracts is usually performed by high performance liquid chromatography with various detector modes, coupled with MS or MS-MS (HPLC-MS or HPLC-MS-MS). High performance thin layer chromatography (HPTLC) has been punctually employed as well as GC after acetylation and/or silylation steps.

Otherwise, we developed a computerized method at the University of Corsica that allows the identification of individual components of a natural mixture (essential oil, vegetable oil, bio-oil, and solvent extract) starting from its ^13^C-NMR spectrum. In a second step, quantitation of some compounds could be performed by ^13^C-NMR or by ^1^H-NMR. The aim of the present study was to investigate the composition of leaf extracts from Corsican *Ilex aquifolium* L., using various NMR methods.

*I. aquifolium*, which is also known as common holly, is a shrub or a small tree with persistent foliage. Its glowing dark green leaves serve as an ornament for Christmas. Flowers that are small and white or pink and odoriferous appear between April and July. Fruits are red berries, about 8 mm diameter, that grow during the August-December period. In the folk medicine, leaves of *I. aquifolium* have been used for their therapeutic properties. They are considered as an antipyretic, astringent, and diuretic. They were also used to treat intermittent fevers, rheumatisms, or as expectorant agents [1,2]. Otherwise, leaves and fruits of *I. aquifolium* have been used to treat cancers of liver, stomach and intestines as well as jaundice and malaria [3,4]. Various *I. aquifolium* leaf extracts (hexane, chloroform, ethyl acetate, and ethanol) displayed moderate antibacterial activities [2]. However, *I. aquifolium* displays a high level of toxicity. For instance, it is reported that children have been poisoned after ingesting common holly fruits and had symptoms such as diarrhea, vomiting, and gastro-intestinal disorders caused by a cyano-glycoside, 2-β-d-glucopyranosyloxy-*p*-hydroxy-6,7-dihydromandelonitrile [5].

Several papers reported on the identification of compounds responsible for the biological activity of *I. aquifolium* extracts. Phytochemicals that belonged to various families have been isolated and identified: amino-acids, saccharides, carotenoids [1], phenol derivatives [1,3,6], fatty acids [1,7], flavonoids [8], and anthocyanes [9]. Lastly, the occurrence of various triterpenes has been reported, such as α-amyrin, β-amyrin, uvaol, erythrodiol, and two triterpene acids such as oleanolic acid and ursolic acid [1,7].

Various studies carried out on oleanolic acid (OA) and/or ursolic acid (UA) demonstrated a wide range of biological activities that have been reviewed including antibacterial, antifungal, anti-tumoral, antiviral, anti-inflammatory, anti-oxidant, anti-cancer, anti-hepatotoxic, anti-hyperglycemic, antileucemic, antimutagene, antipyretic, antidiabetic, and anti-hypertension activities [10,11,12,13,14,15]. Furthermore, UA also possesses an anti-wrinkle activity comparable to that of retinoids, and, therefore, it is appreciated in domains of cosmetic and oncology areas [16].

With respect to their biological and therapeutic activities, identification and quantitation of UA and OA in plant extracts have been investigated following the same scheme. Practically in all papers, one or both acids were first isolated using repetitive chromatography and identified by comparison with spectroscopic data reported in the literature [17]. Then, quantitation of both acids in the plant extract was achieved by chromatographic techniques reviewed by Xu et al. [18]. Recent examples are reported below.

- HPLC (or reversed-phase RP-HPLC) with various detector modes (UV, evaporative light scattering detection, photodiode array detection) appeared as the most employed technique for quantitation of UA and/or OA in plant extracts from *Lantana camara* [19], *Salvia chinensis* [20], *Thymus* ssp [21], and *Nyctanthes arbor-tristis* [22].
- Otherwise, co-quantitation of OA and UA was achieved by LC-MS in oregano (*Origanum* spp.) [23] and in virgin olive oil [24]. An improved scheme involving quantitation using an LC-MS-MS procedure preceded by a qualitative screening using silylation and GC-MS was developed [25].
- HPTLC has been employed in a few studies for quantitation of UA and/or OA in extracts of, inter alia, *Lantana camara* [26] and *Eucalyptus globulus* [27].
- Lastly, both acids have been quantified by GC (FID) after silylation and acetylation steps in plant extracts and in commercial botanicals used as food supplement ingredients [7,28].
- Apart from chromatographic techniques, NMR has been punctually used for quantitation of UA and/or OA in plant extracts. OA has been quantified in a precipitate obtained during the industrial extraction of the leaves of *Olea europaea* using ^13^C-NMR spectroscopy [29]. Otherwise, the combined use of proton-carbon heteronuclear single-quantum coherence (HSQC) and proton-carbon heteronuclear multiple-bond correlation (HMBC), allowed the identification and quantitation of OA and UA in plant extracts of the Lamiaceae and Oleaceae family [30].

It appears from this review of the literature that solvent extracts of *I. aquifolium* display a therapeutic interest useful in various domains such as microbiology, cosmetics, and oncology. Among phytochemicals contained in *I. aquifolium* extracts, UA and OA play an important role.

Identification and quantitation of the two triterpene acids in various plant extracts have been undertaken as follows: isolation of each acid by repeated chromatography, identification by spectroscopic techniques, and then quantitation by HPLC or TLC and punctually GC-MS after derivatization [18]. All these techniques are known to give accurate results but they are also time-consuming.

In the course of our on-going work concerning the valorization of medicinal and aromatic plants growing wild in Corsica, we got interested by *I. aquifolium* that is abundant in Corsican forests, and the possibility to use it as a source of UA and/or OA. Therefore, in order to appreciate the contents of UA and OA produced by *I. aquifolium*, we drew the following scheme based on a novel approach by combining (i) the identification of neutral and acid triterpenes present in extracts from leaves of *I. aquifolium* by combination of column chromatography (CC) and ^13^C-NMR without isolation or purification of the compounds, following an in-house procedure and (ii) quantitation of UA and OA by ^1^H-NMR. The technique is not yet investigated for the two acids, but it allows a short time of occupancy of the spectrometer.

## 2. Results and Discussion

Leaves of *I. aquifolium* harvested in Corsica have been successively extracted with hexane and then with dichloromethane. This two-step procedure was initiated in order to separate as well as possible medium polar triterpenes (esters, aldehydes, and alcohols) from more polar triterpenes (acids). Every extract has been submitted to ^13^C-NMR analysis and then chromatographed on silica gel. All fractions of chromatography were, in turn, analyzed by the same method.

**Figure 1:**
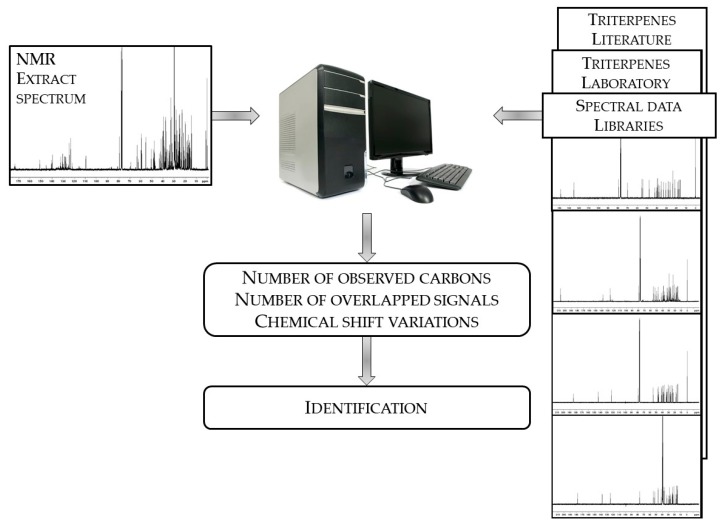
Method for identification of components in solvent extract from leaves of *Ilex aquifolium*.

^13^C-NMR analysis of triterpene mixtures has been done following a computerized procedure initiated and developed at the University of Corsica. This experimental procedure was successfully employed for identifying and quantifying neutral triterpenes and triterpene acids in a dichloromethane extract of cork (*Quercus suber*) [31], and in a hexane extract from leaves of *Olea europea* [29]. In short, identification of the components was carried out by comparing chemical shift values in the mixture spectrum with those of reference spectra compiled in two libraries devoted to triterpenes, using in-house software (Figure 1). The first spectral data library was constructed with spectra recorded in the laboratory while the second one was built with spectral data picked up in recent literature (spectra recorded with diluted samples). Each compound was identified by taking into account three parameters that were directly available from the computer program: (i) the number of observed signals with respect to what was expected, (ii) the difference between the chemical shift of each signal in the mixture and in the reference (Δδ), and (iii) the number of overlapping signals of carbons belonging to two components that fortuitously possess the same chemical shifts [29,31]. If necessary, the occurrence of “false positive” could be avoided by taking into account the relative intensities of the non-overlapped signals of protonated carbons of a given component. A component is considered identified if 50% of its signals are observed and they belong to that compound.

### 2.1. Phytochemicals Contained in I. aquifolium Leaf Extracts

#### 2.1.1. Hexane Extract from Leaves of *I. aquifolium*

**Figure 2:**
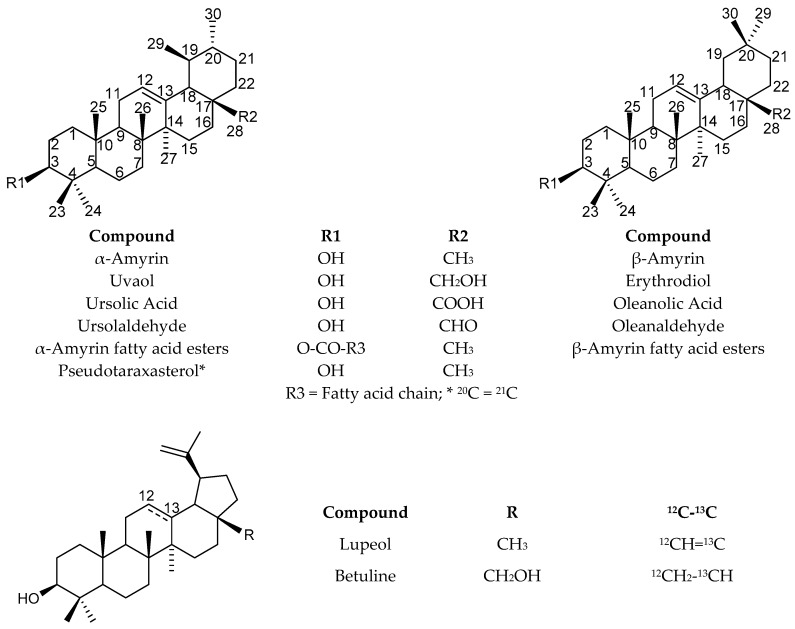
Structures of pentacyclic triterpenes contained in leaves of *I. aquifolium* extracts.

Leaves of *I. aquifolium*, previously crushed in liquid nitrogen, have been extracted with boiling hexane. Solvent has been removed under reduced pressure leading to the hexane extract with a 1.9% yield (*w*/*w* vs. dry material). Analysis of the crude extract by ^13^C-NMR (CDCl_3_) led to the identification of four oxygenated triterpenes: α-amyrin, β-amyrin, lupeol, and uvaol (Figure 2, Table S1). It could be pointed out that, despite the similarity of the structures, their identification does not suffer any doubt since, for every compound, two-thirds of the observed signals belonged solely to that compound.

In the NMR spectrum of the crude hexane extract, various signals with appreciable intensities remained unassigned: (i) two signals around 170 ppm accompanied by two signals around 80 ppm, (ii) a series of signals at approximately 14.1 ppm, 22.7 ppm, 29.7 ppm, and 31.9 ppm characteristic of saturated fatty acid chains coupled with signals at 128–132 ppm belonging to unsaturated fatty acid chains [32,33]. These chemical shift values are associated with the identification of α-amyrin and β-amyrin, which suggested the occurrence of amyrin derivatives that bear various fatty acid esters at C3. In parallel, the presence of a methine signal (confirmed by the distortionless enhancement by polarization transfer - DEPT - spectrum) at 207.5 ppm suggested the occurrence of an aldehydic compound. Therefore, the crude extract was chromatographed on silica gel, which leads to 12 fractions H1–H12. All fractions were submitted to ^13^C-NMR analysis.

- NMR spectra of fractions H2–H4 displayed the signals of all carbons of α-amyrin and β-amyrin as well as those of saturated and unsaturated fatty acid chains. In order to evaluate the nature of these fatty acid chains, a fraction H2 of CC was submitted to trans-esterification. Basic hydrolysis was followed by methylation (BF_3_-Methanol). The organic layer was submitted to GC (Retention indices) and GC-MS analyses. Five methyl esters have been identified with characteristic chains of palmitic acid (C16:0; 54.1%), linolenic acid (C18:3; 13.2%), oleic acid (C18:1; 11.3%), linoleic acid (C18:2; 10.0%), and stearic acid (C18:0; 3.0%).
- Fraction H5 contained α-amyrin, β-amyrin, and lupeol previously identified in the crude extract as well as pseudotaraxasterol, which is present at a lower extent.
- Fraction H7 contained betuline, erythrodiol, and uvaol where the latter is already identified in the crude extract and also present in fraction H8 where it constituted the major component.
- Fraction H6 contained α-amyrin, β-amyrin, and lupeol as well as β-sitosterol not yet identified. The ^13^C-NMR spectrum of that fraction exhibited a series of signals with appreciable intensities including the aldehydic signal (207.5 ppm). Therefore, the fraction H6 has been chromatographed once again, which leads to sub-fractions H6.1-H6.7. Our attention was focused on fraction H6.3. Its ^13^C-NMR spectrum displayed two series of 30 signals. The chemical shifts of the major component were similar to those of *α*-amyrin with a few exceptions: one signal belonged to a carbon linked to a hydroxyl group while one methyl signal was missing and replaced by the aldehydic signal. Search in the literature led us to ursolaldehyde whose ^13^C-NMR spectrum has been reported by Kim et al. [34]. The second series of 30 signals displayed higher intensities on the spectrum of sub-fraction H6.2. Using the same procedure as above, oleanaldehyde was identified by comparison with the chemical shift values reported by Zhang et al. [35]. All the signals belonging to both molecules were observed. The chemical shift differences (Δδ) measured on the spectrum and those reported in the literature are inferior or equal to 0.12 ppm for most carbons, except for those of aldehydic carbons (about 0.20 ppm). It could be pointed out that, despite the similarities of the structures, only four signals out of 30 of oleanaldehyde overlapped with those of ursolaldehyde.

In the hexane extract of leaves from *I. aquifolium* growing wild in Corsica, the following triterpenes have been identified: α-amyrin, β-amyrin, fatty acid esters of amyrins, lupeol, uvaol, betuline, erythrodiol, pseudotaraxasterol, ursolaldehyde, and oleanaldehyde, as well as a steroid, β-sitosterol (Figure 2). α-Amyrin, β-amyrin, and uvaol have been previously identified in the leaves of *I. aquifolium* of Italian origin [7]. Some fatty acid esters of α-amyrin and β-amyrin have been reported including palmitic acid, linoleic acid, and linolenic acid [36]. Ursolaldehyde and oleanaldehyde have been identified in extract from leaves of *I. aquifolium* from the Netherlands [37]. In contrast, lupeol as well as amyrin oleic acid ester and amyrin stearic acid ester are reported for the first time.

#### 2.1.2. Dichloromethane Extract from Leaves of *I. aquifolium*

Leaves of *I. aquifolium* previously extracted by hexane have been extracted by boiling dichloromethane (Soxhlet). Solvent was removed at reduced pressure, which leads to the extract (yield = 2.3% *w*/*w* vs. dry material). The dichloromethane extract was analyzed by ^13^C-NMR (CDCl_3_). Amyrin esters (α-isomers and β-isomers) have been identified as minor components, accompanied by OA. However, the extract being not completely soluble in CDCl_3_, NMR experiments were performed in deuterated dimethylsulfoxyde (DMSO-*d*_6_). In parallel, the spectra of pure (commercial) OA and UA were also recorded in DMSO-*d*_6_. The chemical shift assignments for these compounds have been reported [17]. The chemical shift values of the two major components of the dichloromethane extract fitted perfectly with those of reference compounds (Table S2). According to the relative intensities of the signals of identified components, the dichloromethane extract of leaves from Corsican *I. aquifolium* contained ursolic acid as a major component, which was followed by oleanolic acid and a few minor components such as α-amyrin and β-amyrin esters and β-sitosterol (Figure 2). The occurrence of UA and OA in leaf extract from *Ilex* species, including *I. aquifolium*, has been previously reported [1,7,37].

Quantitation of neutral and acidic triterpenes, including OA, in various extracts has been achieved in our laboratory using ^13^C-NMR. Although various parameters (such as nature of solvent, concentration and pulse sequence) were optimized, it took about two hours to record a spectrum (4.7 Tesla, 10 mm probe or 9.4 Tesla, 5 mm probe) [29,31]. In parallel, the content of lignans in *Cedrus atlantica* resins has been conducted using ^1^H-NMR with a time of occupancy of the spectrometer at the minute level [38]. Therefore, quantitation of OA and UA in dichloromethane leaf extract from *I. aquifolium* has been undertaken with this last technique.

### 2.2. Quantitative Analysis of Ursolic Acid and Oleanolic Acid in I. aquifolium Dichloromethane Extract Using ^1^H-NMR

Whatever the technique used, quantitation of a given component in a mixture requires the comparison of the area(s) of one (or various) signal(s) of that compound with that of a reference compound (internal calibration) or using calibration straight lines (external calibration). Using NMR spectroscopy, it is possible to quantify a component by integration of one of its signals since the area value is proportional to the number of nuclei. Using internal calibration requires a homogenous answer of selected nuclei of the compound and of internal reference. Two parameters are responsible of the uniform or non-uniform answer of nuclei: (i) longitudinal relaxation time (T_1_) of nuclei and (ii) differences in the Nuclear Overhauser Enhancement (NOE) between selected nuclei of the component and those of the reference.

**Figure 3:**
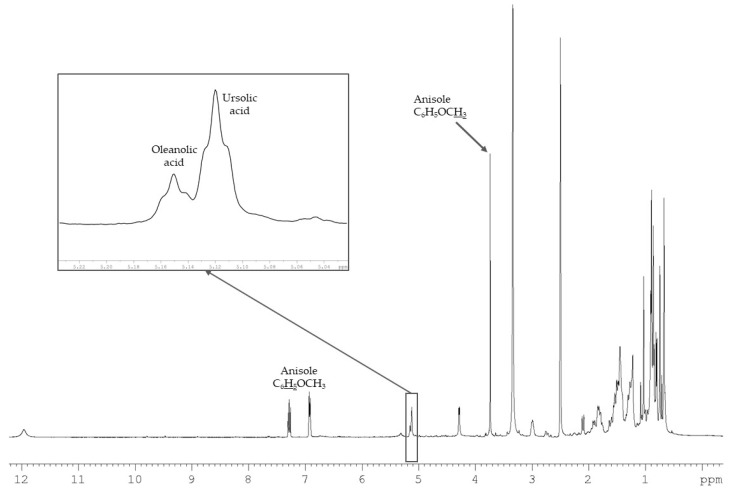
Partial ^1^H-NMR spectrum of *Ilex aquifolium* dichloromethane leaf extract.

Despite the similarity of the structures of UA and OA, it is remarkable that the signals of the ethylenic protons of both acids are sufficiently differentiated (triplets at δ = 5.12 ppm and 5.15 ppm, respectively) (Figure 3). They are sufficiently resolved and they do not overlap with those of minor components. Therefore, they could be used to quantify both triterpene acids in dichloromethane extract. Ursolic acid, which is the main component according to the intensities of signals in the ^13^C-NMR spectrum of the dichloromethane extract, has been chosen as a reference compound for validation of the method. DMSO-*d*_6_ has been chosen as a solvent and anisole (methoxybenzene) as an internal reference. Signals of its methoxy group (3.73 ppm, singlet) as well as those of aromatic protons (6.87 ppm, 6.91 ppm, 7.27 ppm) do not overlap with those of most triterpenes.

T_1_ value of ethylenic proton of ursolic acid has been measured by the inversion recovery method (T_1_ = 0.1 s, DMSO-*d*_6_) and compared to those of the methoxy group of anisole (T_1_ = 0.7 s, DMSO-*d*_6_).

According to Becker et al. [39], we determined and plotted the percentage of recovered signal S:N (%), as a function of the pulse angle α, for the carbons with T_1_ = 0.1 s and 0.7 s (values for selected protons of UA and an internal standard). We observed that this difference is extremely low for a flip angle of 30°.

We checked if the usual pulse sequence used for recording ^1^H-NMR spectra (flip angle = 30°, total recycling time = 3.56 s, see Experimental) was appropriate for quantitation of UA in dichloromethane extract of *I. aquifolium* leaves.

Accuracy, precision, and response linearity of such a procedure was validated by several experiments carried out using commercial UA (purity: 95%) as reported below, while taking into account the relative areas of ethylenic proton of ursolic acid, on the one hand, and the three protons of the methoxy group of anisole, on the other hand. The contents of UA were calculated with Formula (1).

(1) $$m_{UA}=\frac{3\;\times A_{UA}\times M_{UA}\times m_{A}\times P_{A}}{A_{A}\times M_{A}\times P_{UA}}$$

*m_UA_* = calculated mass (mg) of ursolic acid, *A_UA_* and *M_UA_* = area of the ethylenic signal and molecular weight (456 g mol^−1^) of ursolic acid, *m_A_*, *M_A_*, and *A_A_* = amount (mg), molecular weight (108 g mol^−1^), and area of the signal of methoxy group of anisole, fixed at 1.0000 in all experiments. Factor 3 is due to the integration of the three protons of the methoxy group of anisole. *P_A_* and *P_UA_* = purity of anisole (0.99) and UA (0.95), respectively.

Accuracy of the quantitative procedure was checked by comparing the amounts of pure UA, diluted in DMSO-*d*_6_, at different concentrations (1.4–30.9 mg of UA in presence of 1.2 mg of anisole, in 0.5 mL DMSO-*d*_6_) and measured by NMR, with the weighted ones (Table 1). The relative error ranged between −1.0% and +1.7%, which is acceptable for a spectroscopic method.

In order to check the precision, three spectra of the same sample containing ursolic acid and anisole have been recorded with the same experimental conditions. The mass of UA has been calculated using Formula (1). The measured area of UA ethylenic proton with respect to that of the methoxy group of anisole, fixed at 1.0000, were practically constant (1.3006–1.3151). The relative error between the calculated mass of UA and the weighted one varied between −1.0% and 0.0% (Table 2).

**Figure 4:**
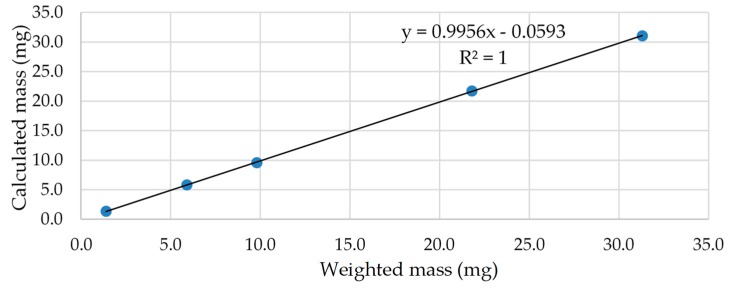
Response linearity of quantitation of ursolic acid using ^1^H NMR.

Response linearity has been evaluated by plotting the measured mass of ursolic acid versus the weighted one in the diagram below and it looks perfectly correct with R^2^ = 1 (Figure 4).

Lastly, the limit of detection (LOD) and the limit of quantitation (LOQ) (see Experimental) have been measured at 0.14 mg and 2.37 mg, respectively.

The quantitation of ursolic acid using ^1^H-NMR being validated, this technique has been used to evaluate the content of UA in *I. aquifolium* dichloromethane leaf extract and it has been extrapolated to OA, which is a structurally close isomer (Table 3, Figure 2). UA accounted for 55.3% of the dichloromethane extract, followed by OA, 20.8%. Both triterpene acids represented more than two thirds of the extract. Therefore, the investigated leaves from *I. aquifolium* contained 1.3% of ursolic acid and 0.5% of oleanolic acid.

For comparison with our results, UA and OA have been found in various plant extracts.

- OA is widely distributed in food and plants. Medicinal plants such as *Lantana camara* are rich sources of OA (0.21–0.58% in flowers, 1.14–1.67% in roots) [26]. Common culinary spices (thyme, clove plants) and fruit plants (apple, loquat, elderberry, and sage) are also sources of OA [40]. Overall, OA can easily be obtained in high yield (up to 3.1%) from olive tree leaves, which is its main commercial source [40].
- In parallel, UA is widely distributed especially in higher plants [41]. For instance, it is present in bearberry leaves (1.24%) [40], in dry flowers of loquat tree (0.22–0.27%) [42], in apple peels (1.43% [40]; 0.71% [43], at around 50 mg per medium sized fruit) [44]). UA has been also found in the leaves of *Ilex paraguariensis* (0.26%) [45]. Concerning *I. aquifolium*, leaves collected in winter near Camaiore (Lucca, Italy) were extracted successively with light petroleum ether and acetone. UA and OA were the principal constituents of the acetone extract and their relative amounts (%) were evaluated by GC of the corresponding methyl esters trimethylsilyl ethers. UA and OA accounted for 3.34% and 0.067%, respectively, with respect to the dried leaves. However, true quantitation was not performed [7].

## 3. Material and Methods

### 3.1. Plant Material and Solvent Extractions

Leaves from *I. aquifolium* have been harvested in December 2017, in the Isolacciu di Fium’Orbu forest (North-East Corsica, France) and kept at −60 °C. Leaves (98 g) have been crushed in liquid nitrogen and then successively extracted with hexane (600 mL, 18 h, Soxhlet apparatus) and dichloromethane (600 mL, 5 h, Soxhlet apparatus). Solvent has been removed under reduced pressure yielding 1.836 g of hexane extract and 2.281 g of dichloromethane extract, respectively.

### 3.2. Column Chromatography on Silica Gel (CC)

#### 3.2.1. Hexane Extract

An aliquot (0.997 g) of hexane extract from *I. aquifolium* leaves has been chromatographed on silica gel (63–200 μm, 60 Å, 25 g), with a gradient of solvent (pentane/diethyl Ether from 100/0 to 0/100), yielding 12 fractions (H1–H12): H1 (100/0; 24 mg), H2 (100/0; 239 mg), H3 (75/25; 17 mg), H4 (75/25; 55 mg), H5 (75/25; 209 mg), H6 (50/50; 95 mg), H7 (50/50; 113 mg), H8 (25/75; 46 mg), H9 (25/75; 17 mg), H10 (0/100; 6 mg), H11 (0/100; 8 mg), and H12 (0/100; 71 mg).

Fraction H6 (84 mg) has been chromatographed once more over silica gel (35–70 μm, 4.0 g) with a gradient of solvent (pentane/diethyl Ether from 60/40 to 0/100) yielding seven sub-fractions (H6.1–H6.7): H6.1 (60/40; 14 mg), H6.2 (60/40; 15 mg), H6.3 (60/40; 14 mg), H6.4 (60/40; 6 mg), H6.5 (50/50; 8 mg), H6.6 (25/75; 6 mg), and H6.7 (0/100; 5 mg).

#### 3.2.2. Dichloromethane Extract

An aliquot (1.075 g) of dichloromethane extract from *I. aquifolium* leaves has been chromatographed on silica gel (63–200 μm, 60 Å, 25 g), with a gradient of solvent (pentane/diethyl Ether from 100/0 to 0/100) yielding nine fractions (D1–D9): D1 (98/2; 215 mg), D2 (92/8; 50 mg), D3 (80/20; 16 mg), D4 (50/50; 69 mg), D5 (50/50; 36 mg), D6 (0/100; 133 mg), D7 (0/100; 129 mg), D8 (0/100; 108 mg), and D9 (0/100; 62 mg).

Fraction D6 (78 mg) has been again chromatographed over silica gel (35–70 μm, 5 g) with a gradient of solvent (pentane/diethyl Ether from 75/25 to 0/100) yielding six sub-fractions (D6.1–D6.6): D6.1 (75/25; 4 mg), D6.2 (75/25; 6 mg), D6.3 (75/25; 16 mg), D6.4 (80/20; 10 mg), D6.5 (80/20; 9 mg), and D6.6 (0/100; 1 mg).

### 3.3. Hydrolysis of α-Amyrin and β-Amyrin Esters. Synthesis of Fatty Acid Methyl Esters

An aliquot of fraction H2 (103 mg) has been added to a solution of ethanolic potassium hydroxide (100 mg KOH/2 mL EtOH) and refluxed for 1.5 h. Then, brine (6 mL) has been added and the mixture extracted with pentane (2 × 10 mL). The aqueous layer has been acidified with HCl (1.2 M) until pH = 1 and extracted with pentane (2 × 10 mL). The organic layer has been dried on anhydrous MgSO_4_ and filtered. The solvent has been removed and yielded 41 mg of fatty acids. Fatty acids have been subjected to BF_3_/Methanol (5 mL) esterification. Brine (20 mL) was added and the mixture was extracted with pentane (3 × 10 mL). The organic layer has been dried on anhydrous MgSO_4_ and filtered. The solvent was removed under reduced pressure and yielded 36 mg of fatty acid methyl esters, which have been subjected to GC and GC-MS analysis (see below).

### 3.4. Analytical GC

Analyses were carried out with a Clarus 500 Perkin Elmer apparatus equipped with two flame ionization detectors (FID), and two fused-silica capillary columns (50 m, 0.22 mm i.d., film thickness 0.25 μm), BP-1 (polydimethylsiloxane), and BP-20 (polyethylene glycol). The oven temperature was programmed from 60 °C to 220 °C at 2 °C/min and then held isothermal at 220 °C for 20 min. The injector temperature was 250 °C. The detector temperature was 250 °C. The carrier gas was hydrogen (1.0 mL/min). The split was 1/60 and the injected volume was 0.5 μL. The relative contents of the oil constituents were expressed as a percentage obtained by peak-area normalization without using correction factors.

### 3.5. GC-MS Analysis

The fatty acid methyl esters were analyzed with a Perkin–Elmer TurboMass detector (quadrupole), directly coupled to a Perkin–Elmer Autosystem XL equipped with a fused-silica capillary column (50 m, 0.22 mm i.d., film thickness 0.25 μm), BP-1 (polydimethylsiloxane). The carrier gas was helium at 0.8 mL/min. The split was 1/75 and the injection volume was 0.5 μL. The injector temperature was 250 °C. The oven temperature was programmed from 60 °C to 220 °C at 2 °C/min and then held isothermal (20 min). The ion source temperature was 250 °C. The energy ionization was 70 eV. The electron ionization mass spectra were acquired over the mass range 40–400 Da.

### 3.6. NMR Spectroscopy

All NMR spectra were recorded on a Bruker AVANCE 400 Fourier Transform spectrometer, operating at 400.132 MHz for ^1^H and 100.623 MHz for ^13^C, equipped with a 5-mm probe, in deuterated chloroform (CDCl_3_) or deuterated dimethylsulfoxide (DMSO-*d*_6_), with all chemical shifts referred to as internal tetramethylsilane (TMS) for spectra recorded in CDCl_3_ and to the signal of solvent (39.39 ppm for ^13^C and 2.50 ppm for ^1^H) for spectra recorded in DMSO-*d*_6_.

^13^C-NMR spectra of extracts and fractions of chromatography were recorded with the following parameters: flip angle 45°, acquisition time, 2.66 s for 128 K data table with a spectral width of 25,000 Hz (250 ppm), relaxation delay D_1_ = 0.1 s (total recycling time = 2.76 s), CPD mode decoupling, and digital resolution, 0.183 Hz/pt. The number of accumulated scans was 1000–3000, depending on the available amount of solvent extract or fraction of chromatography (1–50 mg of mixture in 0.5 mL of solvent). Solvent: CDCl_3_ for extracts (hexane and dichloromethane) and fractions of CC (H1–H12, H6.1–H6.7 and D1–D4), DMSO-*d*_6_ for dichloromethane extract, and fractions D5–D9 of CC.

DEPT spectra were recorded with the same parameters, except flip angle (135°).

^1^H-NMR spectra for quantitation of UA and OA were recorded with the following parameters: flip angle 30°, acquisition time, 2.56 s for 32 K data table with a spectral width of 6410 Hz (16 ppm), and relaxation delay D_1_ = 1 s (total recycling time = 3.56 s).

Longitudinal relaxation delays: The T_1_ values of the ^1^H nuclei were determined by the inversion-recovery method, using the standard sequence: 180°-τ-90°-D_1_, with a relaxation delay D_1_ of 5 s for ^1^H. Each delay of inversion (τ) was, thus, taken into account for the computation of the corresponding T_1_ using the function I_p_ = I_0_ + p.e^−^^τ/T1^

Limits of detection and quantitation: The limits of detection (LOD) and quantitation (LOQ) were determined experimentally using signal-to-noise ratio (S:N). According to Cerceau et al. [46], the concentration for S:N = 10 and for S:N = 150 were set as LOD and LOQ, respectively. Appropriate mass of ursolic acid (1.4–30.9 mg) was introduced in the NMR tube. In addition, 0.5 mL of DMSO-*d*_6_ was added to each of these tubes. Spectra were recorded with 16 scans.

### 3.7. Identification of Individual Components

Both extracts (hexane and dichloromethane) as well as all the fractions of CC were analyzed by ^13^C-NMR, following a computerized procedure developed at the University of Corsica (see results) [30,32]. Neutral triterpenes and triterpene acids were identified by comparing their carbon chemical shifts with those of references compiled in the in-house library (which contains about 50 compounds bearing steroidal structures or pentacyclic structures of oleanane, ursane, or lupane families and that is continuously enriched) or literature data (about 550 compounds). Carbon chemical shifts of amyrin fatty acid esters were compared with literature data [35,36]. Fatty acid methyl esters were analyzed by GC (FID), GC-MS, and ^13^C-NMR by comparing retention indices (RIs), mass spectra, and carbon chemical shifts with those of references compiled in the in-house libraries or literature data.

## 4. Conclusions

In conclusion, leaves of *I. aquifolium* may be considered a source of ursolic acid while oleanolic acid may be considered a side-product. The plant is abundant on the one hand. Non polar and medium-polar components may be almost completely eliminated by a first extraction with hexane. Biologically active compounds, ursolic acid, and oleanolic acid could be easily extracted with dichloromethane and their contents in the extract have been measured, for the first time, by ^1^H-NMR spectroscopy. Taking into account the various biological activities of ursolic acid, reported in the folk medicine and in recent studies (such as antibacterial, antifungal, antiviral, anti-inflammatory, anti-oxidant, anti-wrinkle, anticancer, anti-hepatotoxic, antipyretic, and antidiabetic), and the fair content of that compound in the leaves of *I. aquifolium*, we can assume that this plant could be appreciated in domains of pharmacology, pharmacognosy, cosmetology, and oncology.

Otherwise, NMR spectroscopy appeared as a powerful tool for (i) the identification of individual components of solvent extracts from plants (^13^C-NMR) (ii) quantitation of biologically active compounds (^1^H-NMR) with a short time of occupancy of the spectrometer that allows us to use this technique for quality control.

## Figures and Tables

**Table 1:** Accuracy of ursolic acid (UA) measurements by NMR.

Exp n°	Anisole Area	Mass of Anisole (mg)	UA Area	Weighted Mass (mg)	Calculated Mass (mg)	RE%
1	1.0000	1.2	0.0857	1.4	1.4	0.0
2	1.0000	1.2	0.3782	5.9	6.0	1.7
3	1.0000	1.2	0.6187	9.8	9.8	0.0
4	1.0000	1.2	1.3006	20.8	20.6	−1.0
5	1.0000	1.2	1.9297	30.9	30.6	−1.0

UA = Selected signal of UA at 5.12 ppm, calculated mass, according to Formula (1); RE: relative error.

**Table 2:** Precision of ursolic acid (UA) measurements by NMR.

	Test 1	Test 2	Test 3
Weighted mass of UA (mg)	20.8	20.8	20.8
UA Area	1.3006	1.3151	1.3077
Calculated mass of UA (mg)	20.6	20.8	20.7
RE (%)	−1.0	0.0	−0.5

UA Area: area of the signal of the ethylenic proton of UA with respect to that of methoxy protons of anisole fixed at 1.0000; RE: relative error.

**Table 3:** Quantitation of ursolic acid (UA) and oleanolic acid (OA) in the dichloromethane leaf extract from *Ilex aquifolium.*

Ursolic Acid		Oleanolic Acid	
A_UA_	0.4380	A_OA_	0.1658
m_UA_ (mg)	8.7	m_OA_ (mg)	3.3
UA content (%)	55.3	OA content (%)	20.8

Mass of extract: 15.9 mg. Area of the signal of methoxy group of anisole is fixed at 1.0000 in all experiments, amount of anisole (m_A_) = 1.5 mg, purity of anisole: 0.99, molecular weight of UA and OA: 456 g mol^−1^, molecular weight of anisole: 108 g mol^−1^, A_UA_ and A_OA_: areas of the signal belonging to the ethylenic proton of UA (5.12 ppm) and of OA (5.15 ppm), respectively, m_UA_ and m_OA_: calculated mass (mg) of UA and OA, respectively, according to Formula (1).

## Supplementary Materials

The following are available online. Table S1: Listing of the chemical shift values compiled in the ^13^C-NMR spectrum of the *Ilex aquifolium* hexane extract allowing the identification of α-amyrin, β-amyrin, lupeol and uvaol., Table S2: Listing of the chemical shift values compiled in the ^13^C-NMR spectrum of the *Ilex aquifolium* dichloromethane extract allowing the identification of ursolic and oleanolic acids.
