# Supplementary material for: Identification and Quantitation of Ursolic and Oleanolic Acids in Ilex aquifolium L. Leaf Extracts Using 13C and 1H-NMR Spectroscopy
Source: Molecules. 2019 Dec 3;24(23):4413. doi: 10.3390/molecules24234413 (PMC6930589; doi:10.3390/molecules24234413)
Supplement: Supplementary file 1 [file molecules-24-04413-s001.pdf]

Table S1. Listing of the chemical shift values compiled in the ^{13}C NMR spectrum of the *Ilex aquifolium* hexane extract allowing the identification of α-amyrin, β-amyrin, lupeol and uvaol.

Peaks	Chemical shifts δ (ppm, CDCl3)	Relative intensities	Compounds	Reference chemical shifts δ (ppm); Laboratory library
1	207.48	0.99		
2	173.73	3.63	*α-/β-Amyrin fatty acid ester*	
3	173.68	1.68	*α-/β-Amyrin fatty acid ester*	
4	150.91	1.83	Lupeol	150.87
5	145.18	0.92	β-Amyrin	145.21
6	139.60	4.17		
7	139.57	2.80	α-Amyrin	139.62
8	138.69	1.98	Uvaol	138.75
9	135.17	2.69		
10	131.93	4.33	*Fatty acid ester chains*	
11	130.25	2.27	*Fatty acid ester chains*	
12	130.23	2.54	*Fatty acid ester chains*	
13	130.19	2.46	*Fatty acid ester chains*	
14	130.03	1.45	*Fatty acid ester chains*	
15	129.97	1.67	*Fatty acid ester chains*	
16	129.74	1.35	*Fatty acid ester chains*	
17	128.26	4.09	*Fatty acid ester chains*	
18	128.24	3.62	*Fatty acid ester chains*	
19	128.02	1.60	*Fatty acid ester chains*	
20	127.90	1.47		
21	127.72	2.84		
22	127.70	2.44		
23	127.10	4.07		
24	126.19	0.74		
25	125.01	7.34	Uvaol	125.06
26	124.40	5.78	α-Amyrin	124.46
27	124.31	6.54		
28	124.26	1.50		
29	122.34	0.87		
30	121.71	1.39	β-Amyrin	121.76
31	121.63	1.23		
32	109.33	2.17	Lupeol	109.28
33	80.82	0.56	*α-/β-Amyrin fatty acid ester*	
34	80.61	5.38	*α-/β-Amyrin fatty acid ester*	
35	79.06	5.25	α-Amyrin/β-Amyrin	79.08/79.06
36	79.02	7.52	Lupeol /Uvaol	78.98/79.03
37	77.33	72.34	CDCl3	

38	77.22	2.79		
39	77.02	73.63	CDCl3	
40	76.70	72.66	CDCl3	
41	74.48	0.71		
42	69.89	2.52	Uvaol	69.93
43	62.09	0.57		
44	59.05	10.14	α-Amyrin	59.11
45	55.29	4.00	Lupeol	55.28
46	55.24	8.10	α-Amyrin	55.23
47	55.17	7.35	β-Amyrin/Uvaol	55.22/55.21
48	54.01	3.79	Uvaol	54.06
49	52.61	1.07		
50	50.42	3.19	Lupeol	50.42
51	50.31	0.69		
52	50.13	1.01		
53	48.29	3.27	Lupeol	48.29
54	47.97	2.78	Lupeol	47.98
55	47.70	5.37	α-Amyrin	47.76
56	47.64	4.94	β-Amyrin	47.70
57	47.62	8.26	Uvaol	47.68
58	47.55	2.42		
59	47.21	2.82	β-Amyrin	47.27
60	46.81	1.36	β-Amyrin	46.90
61	46.77	1.15		
62	46.45	0.70		
63	45.82	1.00		
64	43.90	0.56		
65	42.98	2.70	Lupeol	43.00
66	42.81	2.56	Lupeol	42.83
67	42.32	1.23		
68	42.14	0.81		
69	42.05	7.48	α-Amyrin	42.12
70	42.01	3.00	Uvaol	42.07
71	41.69	2.32	β-Amyrin	41.76
72	41.52	10.96	α-Amyrin	41.56
73	41.34	0.82		
74	41.06	0.89		
75	40.81	2.24	Lupeol	40.83
76	40.01	7.31	α-Amyrin/Uvaol	40.06/40.04
77	39.99	8.67	Lupeol	40.01
78	39.86	1.33		
79	39.79	3.07	β-Amyrin	39.83

80	39.72	2.62		
81	39.64	9.37	α-Amyrin	39.69
82	39.60	13.81	α-Amyrin	39.64
83	39.49	0.93		
84	39.41	4.48	Uvaol	39.45
85	39.36	4.20		
86	39.34	4.46	Uvaol	39.38
87	39.04	0.90		
88	38.98	1.51		
89	38.83	4.21	α-Amyrin/β-Amyrin	38.83/38.84
90	38.78	8.98	α-Amyrin/ Lupeol /Uvaol	38.81/38.80/38.82
91	38.75	9.68	Lupeol /Uvaol	38.79/38.80
92	38.70	4.45	β-Amyrin	38.63
93	38.58	2.28		
94	38.43	6.45		
95	38.23	1.19		
96	38.04	3.76	Lupeol	38.05
97	37.97	3.18	Uvaol	38.03
98	37.82	0.99		
99	37.73	6.78		
100	37.42	2.82		
101	37.35	1.59		
102	37.28	2.94	β-Amyrin	37.20
103	37.13	3.42	β-Amyrin/ Lupeol	37.05/37.15
104	36.91	2.12		
105	36.88	4.30	α-Amyrin	36.93
106	36.85	3.59	Uvaol	36.90
107	36.78	6.69		
108	36.66	1.55		
109	36.14	0.67		
110	36.06	0.86		
111	35.57	3.09	Lupeol	35.59
112	35.17	3.41	Uvaol	35.20
113	34.85	6.58	β-Amyrin	34.77
114	34.72	2.25		
115	34.66	4.97		
116	34.51	1.95		
117	34.27	3.40	Lupeol	34.28
118	34.07	1.29		
119	34.00	1.28		
120	33.93	1.38		
121	33.82	1.24		

122	33.73	9.23	α-Amyrin	33.78
123	33.33	2.33	β-Amyrin	33.35
124	33.18	0.97		
125	33.09	1.13		
126	32.92	5.12	α-Amyrin	32.98
127	32.85	6.20	Uvaol	32.86
128	32.80	4.76	β-Amyrin	32.70
129	32.69	2.72		
130	32.64	1.80		
131	32.57	1.45		
132	32.47	2.68	β-Amyrin	32.52
133	32.34	1.03		
134	32.19	4.12	*Fatty acid ester chains*	
135	31.92	17.53	*Fatty acid ester chains*	
136	31.85	1.85	*Fatty acid ester chains*	
137	31.58	13.95	*Fatty acid ester chains*	
138	31.52	3.13	*Fatty acid ester chains*	
139	31.24	8.30	α-Amyrin	31.29
140	31.07	2.73		
141	30.93	1.02	β-Amyrin	31.01
142	30.61	3.44	Uvaol	30.65
143	30.17	1.06		
144	29.83	4.07	*Fatty acid ester chains*	
145	29.70	100.00	Lupeol/*Fatty acid ester chains*	29.73
146	29.66	32.29	*Fatty acid ester chains*	
147	29.58	15.18	*Fatty acid ester chains*	
148	29.52	4.77	*Fatty acid ester chains*	
149	29.47	10.53	*Fatty acid ester chains*	
150	29.36	18.73	*Fatty acid ester chains*	
151	29.32	6.18	*Fatty acid ester chains*	
152	29.26	9.49	*Fatty acid ester chains*	
153	29.16	14.80	*Fatty acid ester chains*	
154	29.11	8.91	*Fatty acid ester chains*	
155	29.05	5.74	*Fatty acid ester chains*	
156	28.74	12.58	α-Amyrin	28.77
157	28.39	2.85	β-Amyrin	28.41
158	28.24	1.38		
159	28.08	17.10	α-Amyrin (x2)/β-Amyrin/Uvaol	28.15 (x2)/28.12/28.14
160	27.97	8.00	Lupeol	28.01
161	27.66	1.01		
162	27.59	1.32		
163	27.43	3.42	Lupeol (x2)	27.43 (x2)

164	27.36	3.80	β-Amyrin	27.28
165	27.19	14.65	α-Amyrin/Uvaol	27.21/27.28
166	27.02	1.06		
167	26.90	9.25	β-Amyrin	26.98
168	26.86	1.55		
169	26.76	1.46		
170	26.59	7.31	α-Amyrin	26.66
171	26.38	4.18	β-Amyrin	26.29
172	26.14	1.77		
173	25.98	5.13	Uvaol	26.04
174	25.95	2.16	β-Amyrin	26.01
175	25.73	1.43		
176	25.68	1.48		
177	25.60	7.59	*Fatty acid ester chains*	
178	25.52	6.52		
179	25.27	4.51		
180	25.16	7.12		
181	25.14	5.73	Lupeol	25.15
182	24.85	1.50		
183	24.79	3.47		
184	24.44	1.67		
185	24.21	1.06		
186	24.07	0.97		
187	23.77	1.75		
188	23.68	3.83	β-Amyrin	23.71
189	23.62	6.58		
190	23.52	3.69	β-Amyrin	23.56
191	23.42	5.52		
192	23.36	13.57	Uvaol	23.38
193	23.30	5.37	α-Amyrin/Uvaol	23.36/23.34
194	23.26	7.79	α-Amyrin/Uvaol	23.30/23.33
195	23.22	8.76		
196	23.05	1.12		
197	22.69	18.19	*Fatty acid ester chains*	
198	22.65	14.16	*Fatty acid ester chains*	
199	22.62	5.08	*Fatty acid ester chains*	
200	22.57	2.67		
201	22.08	0.84		
202	21.96	0.95		
203	21.61	0.83		
204	21.39	13.22	α-Amyrin	21.41
205	21.31	4.48	Uvaol	21.32

206	21.07	1.76		
207	21.03	1.67		
208	20.92	3.22	Lupeol	20.95
209	20.75	1.32		
210	20.69	3.12		
211	20.54	3.96		
212	20.43	0.64		
213	19.81	1.04		
214	19.74	2.85		
215	19.64	1.89		
216	19.38	0.70		
217	19.29	3.96	Lupeol	19.34
218	19.02	0.96		
219	18.75	1.57		
220	18.57	0.55		
221	18.34	6.47	α-Amyrin/β-Amyrin	18.39/18.41
222	18.31	7.70	Lupeol	18.37
223	18.28	6.59	Uvaol	18.34
224	17.99	4.01	Lupeol	18.04
225	17.67	1.34		
226	17.49	7.87	α-Amyrin	17.49
227	17.46	6.05	Uvaol	17.36
228	17.34	3.99		
229	17.19	1.20		
230	16.98	0.72		
231	16.85	13.11	α-Amyrin	16.90
232	16.81	8.65	β-Amyrin	16.84
233	16.74	4.44	Uvaol	16.80
234	16.65	1.33		
235	16.56	0.97		
236	16.16	3.37	Lupeol	16.16
237	16.10	3.84		
238	16.02	3.79	Lupeol	16.01
239	15.96	4.04		
240	15.71	7.93	α-Amyrin	15.69
241	15.67	7.21	β-Amyrin/Uvaol	15.65/15.66
242	15.62	8.51	α-Amyrin/β-Amyrin/Uvaol	15.64/15.56/15.63
243	15.53	1.88		
244	15.50	2.91		
245	15.37	4.07	Lupeol	15.42
246	14.53	2.68	Lupeol	14.53
247	14.27	3.94	*Fatty acid ester chains*	

248	14.11	29.02	*Fatty acid ester chains*
249	14.07	2.83	
250	12.23	0.79	
251	11.97	0.79	
252	11.84	0.73	
253	11.76	0.78	
254	11.42	2.23	
255	11.29	0.77	
256	0.00	27.52	

Table S2. Listing of the chemical shift values compiled in the ^{13}C NMR spectrum of the *Ilex aquifolium* dichloromethane extract allowing the identification of ursolic and oleanolic acids.

Peaks	Chemical shifts δ (ppm, DMSO-d6)	Relative intensities	Compounds	Reference chemical shifts δ (ppm); Laboratory library
1	178.41	8.25	Oleanolic acid	178.42
2	178.11	46.42	Ursolic acid	178.14
3	143.68	7.80	Oleanolic acid	143.68
4	138.04	44.79	Ursolic acid	138.05
5	132.43	2.99		
6	127.75	3.90		
7	127.40	2.97		
8	126.80	2.51		
9	124.44	38.65	Ursolic acid	124.44
10	124.02	2.49		
11	121.38	6.35	Oleanolic acid	121.39
12	99.40	3.14		
13	76.69	54.70	Ursolic acid / Oleanolic acid	76.70/76.70
14	65.81	2.55		
15	55.85	3.01		
16	54.66	51.46	Ursolic acid / Oleanolic acid	54.65/54.69
17	52.24	46.87	Ursolic acid	52.24
18	46.96	10.34	Oleanolic acid	46.90
19	46.89	46.43	Ursolic acid	46.89
20	46.68	74.08	Ursolic acid	46.69
21	46.30	2.73		
22	45.54	5.10	Oleanolic acid	45.55
23	45.30	12.35	Oleanolic acid	45.31
24	41.50	76.17	Ursolic acid	41.51
25	41.16	13.46	Oleanolic acid	47.17
26	40.66	7.99	Oleanolic acid	40.66
27	40.01	87.75	Oleanolic acid	40.12
28	39.81	260.51	DMSO-d6	
29	39.60	516.11	DMSO-d6	
30	39.39	605.16	DMSO-d6	
31	39.18	511.20	DMSO-d6	
32	38.97	307.00	DMSO-d6 / Ursolic acid	38.97
33	38.76	83.03	DMSO-d6	
34	38.37	54.26	Ursolic acid	38.37

35	38.31	50.11	Ursolic acid	38.30
36	38.24	100.00	Ursolic acid / Oleanolic acid	38.24/38.24
37	38.11	33.70	Ursolic acid	38.11
38	37.93	5.66	Oleanolic acid	37.94
39	36.61	3.11		
40	36.46	14.77		
41	36.39	81.20	Ursolic acid / Oleanolic acid	36.39/36.39
42	36.18	33.39	Ursolic acid	36.18
43	33.52	2.45	Oleanolic acid	33.53
44	33.20	6.02	Oleanolic acid	33.29
45	32.69	10.23	Oleanolic acid	32.70
46	32.57	32.36	Ursolic acid	32.57
47	32.28	6.39	Oleanolic acid	32.29
48	31.95	7.01		
49	31.18	4.16		
50	30.25	14.56	Oleanolic acid	30.25
51	30.06	29.67	Ursolic acid	30.06
52	28.91	12.03		
53	28.60	5.83		
54	28.44	4.60		
55	28.30	5.82		
56	28.11	70.37	Ursolic acid / Oleanolic acid	28.13/28.08
57	27.95	3.23		
58	27.40	32.74	Ursolic acid / Oleanolic acid	27.41/27.42
59	27.25	4.56		
60	27.06	6.49	Oleanolic acid	27.06
61	26.86	35.37	Ursolic acid	26.86
62	26.49	2.76		
63	25.45	12.75	Oleanolic acid	25.45
64	25.05	3.45		
65	24.96	3.11		
66	24.27	3.01		
67	23.67	33.61	Ursolic acid / Oleanolic acid	23.68/23.68
68	23.22	13.44		
69	23.12	73.12	Ursolic acid / Oleanolic acid	23.14/23.13
70	22.71	35.62	Ursolic acid / Oleanolic acid	22.72/22.72
71	22.47	6.30		
72	22.32	3.15		

73	21.98	4.65		
74	21.09	2.53		
75	20.93	74.17	Ursolic acid	20.94
76	19.90	3.08		
77	17.87	36.45	Ursolic acid / Oleanolic acid	17.87/17.89
78	17.17	2.65		
79	16.86	70.97	Ursolic acid	16.88
80	16.76	72.28	Ursolic acid	16.78
81	16.69	12.49	Oleanolic acid	16.69
82	15.93	79.93	Ursolic acid	15.94
83	15.87	15.61	Oleanolic acid	15.87
84	15.35	2.85		
85	15.08	78.83	Ursolic acid	15.09
86	14.96	13.81	Oleanolic acid	14.96
87	13.80	3.48		
